# Chiropteran (*Hypsugo savii*) Post-Natal Brain 2D-In Vitro Models: Primary Cell Isolation, Immortalization and Transcriptomic Changes

**DOI:** 10.3390/ani16132037

**Published:** 2026-07-02

**Authors:** Antonella Molinari, Valentina Moccia, Massimiliano Babbucci, Luca Peruzza, Enrico Negrisolo, Cinzia Centelleghe, Sandro Mazzariol, Valentina Elena Giuditta Zappulli

**Affiliations:** 1 Department of Comparative Biomedicine and Food Science, University of Padua, 35020 Legnaro, Italy; valentina.moccia@unipd.it (V.M.); massimiliano.babbucci@unipd.it (M.B.); luca.peruzza@unipd.it (L.P.); enrico.negrisolo@unipd.it (E.N.); cinzia.centelleghe@unipd.it (C.C.); sandro.mazzariol@unipd.it (S.M.); valentina.zappulli@unipd.it (V.E.G.Z.); 2 Department of Agronomy, Food, Natural Resources, Animals and Environment, University of Padua, 35020 Legnaro, Italy

**Keywords:** *One Health*, immunity, bat, brain, 2D-*in vitro* models, primary cells, immortalized cells, electroporation, liposome-mediated transfection

## Abstract

Bats are important reservoirs of zoonotic pathogens and valuable models for studying antiviral tolerance and neuroinflammation within a *One Health* framework. However, chiropteran neural 2D-*in vitro* models remain limited. Here, we established and characterized the first chiropteran primary (CpBCs) and immortalized (CiBCs) cell lines from *Hypsugo savii* species. To overcome the limited lifespan of CpBCs, immortalization strategies based on human telomerase reverse transcriptase (hTERT) and Simian virus 40 large T antigen (SV40) were evaluated. Electroporation-mediated transfection with SV40 successfully generated CiBCs, whereas liposome-mediated and hTERT-based approaches were unsuccessful. RNA sequencing revealed marked transcriptional changes comparing CiBCs with CpCBs, such as the upregulation of pathways related to cell cycle progression, DNA replication, and proliferation in CiBCs, together with the downregulation of apoptosis, inflammatory signaling, and immune-related pathways. Immortalized cells also exhibited enrichment of neural stem cell-like and cancer-associated signatures, suggesting partial dedifferentiation induced by SV40-mediated immortalization. Overall, this study provides a novel chiropteran brain-derived 2D-*in vitro* platform for investigating bat neurobiology, host–pathogen interactions, viral tolerance, and neurotropic infectious diseases relevant to emerging zoonoses.

## Simple Summary

Bats play an important role as a natural reservoir of pathogens in nature and can carry viruses that affect humans and other animals without becoming seriously ill themselves. Understanding how bats resist infections and protect their brains from inflammation may help scientists develop new strategies for studying diseases in both animals and people. However, there are still very few laboratory models available to study bat brain cells. In this study, we created the first primary and immortalized brain cell cultures from the bat species *Hypsugo savii*, a common European bat that often lives close to human environments. We tested different methods to produce long-lasting cell cultures and found that a method based on Simian virus 40 successfully generated stable immortalized cells. We also compared the genetic activity of the original and immortalized cells and found important differences related to cell growth, immune responses, and cell survival. These new bat brain cell models provide a valuable tool for studying how bats interact with viruses, how they tolerate infections without severe disease, and how their brains respond to inflammation. The results may support future research on emerging zoonotic diseases and improve our understanding of animal and human health within a *One Health* approach.

## 1. Introduction

The *One Health* framework emphasizes the interconnectedness of human, animal, and environmental health, highlighting the need for integrated approaches to understand and mitigate emerging biological threats [1,2]. Recent global events, particularly the COVID-19 pandemic, have intensified scientific attention toward wildlife species as reservoirs of zoonotic pathogens [1,2,3]. Among these, bats (order *Chiroptera*) have emerged as particularly significant due to their remarkable ecological diversity and unique host–pathogen interactions [1,2]. Several studies suggest that bats possess a “tuned-down” immune system, characterized by constitutive antiviral defenses and a controlled inflammatory profile [1,2,4,5]. Additionally, the high metabolic demands associated with powered flight result in elevated production of reactive oxygen species [1]. Consequently, bats have evolved enhanced DNA repair pathways and antioxidant systems, which mitigate cellular damage and reduce inflammation-associated tissue injury during viral replication [1,2,4,5,6]. Their tendency to form dense roosting colonies, relatively long lifespans for their body size, and capacity for torpor or hibernation promote sustained low-dose pathogen exposure and may favor the evolution of immune tolerance while limiting viral replication dynamics [1,3,4]. Importantly, these adaptations have broader biomedical implications, particularly towards the study of diseases affecting the nervous system (NS) [2,4].

Notably, bats are recognized as natural reservoirs for a wide range of NS zoonotic infections. While some of these agents—particularly Lyssaviruses—can occasionally induce mild pathological manifestations in bats, most do not cause any pathology, especially within the NS [2]. Additionally, regarding NS non-infectious diseases, the dampened inflammatory responses observed in bats are of particular interest for understanding human neuroinflammatory and neurodegenerative disorders such as Multiple Sclerosis, Alzheimer’s disease, and Parkinson’s disease [7,8,9,10,11]. Furthermore, bats exhibit exceptional longevity and maintain cognitive function with age, suggesting enhanced resistance to neurodegeneration and cancer [6,11,12,13]. Intriguingly, bats’ specialized sensory systems, particularly echolocation, rely on highly adapted hippocampal and cortical circuits involved in spatial navigation and sensorimotor integration [1]. These systems might also inform research into neurological conditions such as epilepsy and schizophrenia [11]. Moreover, the metabolic demands of flight expose bats to intermittent hypoxia-like conditions, suggesting potential adaptations relevant to ischemic tolerance in conditions such as stroke and traumatic brain injury [11,14].

Despite their scientific value, bats remain underrepresented as experimental models. Limitations include challenges in breeding and maintenance, limited genetic manipulation tools compared to traditional laboratory organisms, and high interspecies variability [1,2,3]. In this context, standardized 2D-*in vitro* systems may provide a valuable alternative model for improving our understanding of bat biology and physiology.

Primary somatic cells (pSCs), either isolated in-house or commercially available, are widely used in human biomedical research [15]. However, their limited lifespan and susceptibility to replicative senescence restrict long-term applications [15,16]. Therefore, to overcome these limitations, immortalization of pSCs is required to stop cell-aging mechanisms [17,18,19,20,21,22]. In mammals, common genes used to generate immortalized cells employ viral oncogenes—such as Simian virus 40 large T antigen (SV40), human papillomavirus E6/E7, or Epstein–Barr virus latent membrane protein 1—or non-viral oncogenes (e.g., KRAS and MYC), often in combination with telomerase reverse transcriptase (hTERT) expression and/or short-hairpin RNA constructs [15,16,17,18,20,21,22,23,24,25,26,27,28,29,30,31]. Expression by pSCs of one of these genes can be achieved using different methodologies, including biological (e.g., transduction), chemical, or physical approaches, with liposome- and electroporation-mediated transfection being the most commonly used ones [15,16,32,33,34].

In human biomedical research, a broad array of primary NS non-cancerous cell types—including neurons, astrocytes, oligodendrocytes, Schwann cells and microglia—are commercially available together with their immortalized counterparts (except for neurons). Numerous laboratories have also successfully isolated and immortalized human astrocytes, Schwann cells, microglia, and neural stem cells (NSCs), whereas oligodendrocytes remain restricted to pSCs [29,30,35,36,37,38,39,40,41,42,43,44,45]. In contrast, non-tumorigenic NS-derived cells from veterinary species—excluding conventional laboratory animals such as rodents and rabbits—remain limited. Commercially available NS pSCs include canine neurons, astrocytes, and microglia, as well as bovine, feline, and non-human primate astrocytes. Available immortalized NS cells include feline astrocytes, alongside non-characterized mustelid and ovine NS-derived cells. In-house isolations have included porcine neurons, astrocytes, and oligodendrocytes, as well as canine, bovine, caprine, and porcine NSCs [46,47,48,49,50,51,52,53,54,55,56,57]. In addition, immortalization efforts have yielded porcine NSCs and ovine astrocytes and microglia [55,58,59,60]. With specific regard to chiropteran NS 2D-*in vitro* models, several cell lines have been reported, both commercially available and in-house isolated.

In light of the previous considerations, the present study aimed to isolate and establish post-natal chiropteran primary brain-derived cells (CpBCs), herein called brain cells, and their immortalized counterparts (CiBCs) to generate a robust 2D-*in vitro* model that can support investigations into bat-specific antiviral mechanisms, neuroprotection, and NS-related host–pathogen interactions within a *One Health* context. Additionally, we examined for the first time the transcriptomic alterations associated with the immortalization of CpBCs to better characterize the changes underlying this process at the RNA level [2].

## 2. Materials and Methods

### 2.1. Tissue Sampling and DNA Extraction for Species Identification

A young male bat was humanely euthanized due to an irreparable humerus fracture at a specialized veterinary clinic following a thorough clinical evaluation. Necropsy was performed by a trained pathologist immediately after death, and samples were transported to the Department of Comparative Biomedicine and Food Safety of the University of Padua (Legnaro, Italy) for processing.

For species identification, skin, muscle, and liver tissue fragments (approximately 0.5 × 0.5 cm) were immediately collected and stored at −20 °C until DNA extraction using the DNeasy Blood and Tissue Kit (Qiagen, Venlo, The Netherlands, #69506), following the manufacturer’s protocol. Extracted DNA was then quantified using Nanodrop ND-1000 technology (Wilmington, DE, USA). Only high-quality DNA samples obtained from muscle and liver tissues were selected and submitted for species identification through DNA barcoding analysis targeting the mitochondrial gene Cytochrome c oxidase subunit I (COI) [61]. PCR amplifications were performed using the universal primers for COI-barcode [62], and PCR products were sent for sequencing to BMR Genomics Company (Padua, Italy).

### 2.2. Isolation and Establishment of Chiropteran Primary Brain Cells

For the isolation of CpBCs, brain tissue fragments (approximately 0.5 × 0.5 cm) were immediately suspended in sterile phosphate-buffered saline (PBS) supplemented with 10% penicillin–streptomycin (P/S, Thermo Fisher Scientific, Waltham, MA, USA, #15140122). After transport at 4 °C, under sterile conditions, brain biopsies were washed with PBS, dissected to remove visible blood vessels, and minced with a sterile razor blade. Tissue dissociation was then performed using the Papain Dissociation System (Worthington Biochemical Corporation, Lakewood, NJ, USA, #LK003150) following the manufacturer’s protocol. Briefly, samples were digested at 37 °C for 1.5 h, filtered through a 70 µm cell strainer, and centrifuged at 300× *g* for 5 min. The resulting pellet was subjected to density gradient centrifugation with an albumin-ovomucoid inhibitor solution at 100× *g* for 5 min, plated in two wells of a 24-well standard plate (sP1), and cultured in Dulbecco’s Modified Eagle Medium/Nutrient Mixture F-12 (DMEM/F-12, Thermo Fisher Scientific, #11320033) with 1% GlutaMAX™ supplement (Glutamax, Thermo Fisher Scientific, #35050061), 1% MEM Non-Essential Amino Acids solution (NEAA, Thermo Fisher Scientific, #11140050), 1% N-2 supplement (N-2, Thermo Fisher Scientific, #17502048), 2% B-27™ supplement (B-27, Thermo Fisher Scientific, #17504044), 1% P/S, 20 ng/mL of human basic fibroblast growth factor (hFGF, Thermo Fisher Scientific, #100-18B-50UG), and 20 ng/mL of human epidermal growth factor (hEGF, Merck, Darmstadt, Germany, #E9644) (herein as cBCsm1) in an atmosphere of humidified air and 5% CO_2_ at 37 °C. Cytokines were always added fresh to the cell culture media. Additionally, 72 h after isolation, the supernatant from the wells of sP1 was replated in one well each of a 24-well standard plate (sP2) in cBCsm1 supplemented with 2% fetal bovine serum (FBS, Thermo Fisher Scientific, #A5256701) (herein as cBCsm2), and fresh cBCm1 was replaced in all wells of sP1. Furthermore, 48 h after the first replating, fresh cBCsm1 or cBCsm2 was added to all wells. Four days after the first replating, media changes with cBCsm1 or cBCsm1 supplemented with 10% FBS (herein as cBCsm3) were performed for all wells of sP1 and sP2, respectively. The day after, fresh cBCsm1 and cBCsm3 were added to all wells. Eight days after the first replating, media changes with cBCsm1 were performed for sP1 and one well of sP2, while for the other well of sP2, a medium change was performed using cBCsm3. When the day after CpBCs reached nearly 100% confluence in all wells (except for the sP2 well cultured in cBCsm1), cells were single-washed with PBS and enzymatically detached using Accutase (Merck, #A6964) diluted (1:3) in PBS at 37 °C for 5 min. Then, CpBCs were centrifuged at 400× *g* for 5 min and seeded (1:2) into either one well each of a 12-well standard (sP3) or two wells of a 24-well standard plate (sP4) for the wells of sP1 and sP2, respectively. Either cBCsm1 or cBCsm3 was replaced with the leftover cells in sP1 and sP2 wells and changed every other day to sP3 and sP4, respectively. When the cells reached nearly 100% confluence in sP3, CpBCs were enzymatically split using Accutase and seeded into two wells of a 12-well standard (sP5) and in one well of a 24-well standard plate (sP6) in cBCsm1. From this moment on, all wells were cultured in cBCsm3, and subsequently changed the day after splitting and every other day. CpBCs were further expanded in the 6-well standard plate format with a seeding rate ranging from 1:2 to 1:4, according to downstream experiments. cBCsm3 was always replaced the day after splitting and three times per week, following a single wash with PBS when observing large amounts of cellular death and debris. CpBCs were regularly cryopreserved in a freezing medium composed of 90% FBS and 10% dimethyl sulfoxide and screened every three months for mycoplasma contamination using the MycoAlert^®^ Mycoplasma Detection kit (Lonza, Basel, Switzerland, #LT07-703) and the Venor^®^GeM Classic (Minerva Biolabs, Berlin, Germany, #11-1025) kit.

### 2.3. Immortalization of Chiropteran Primary Brain Cells

DH5-alpha *Escherichia coli* bacteria, transduced with either plasmid DNA encoding SV40 (pSV3-neo, ATCC, Manassas, VA, USA, #37150) or hTERT (pCl neo-hEST2, Addgene, Watertown, MA, USA, #1781), were grown overnight at 37 °C in Luria–Bertani medium (LB, Microbiol, Catania, Italy, #70402) agar plates supplemented with 100 μg/mL of ampicillin (Merck, #A5354-10). The day after, single antibiotic-resistant bacterial colonies were picked and allowed to grow on a shaking platform overnight at 37 °C in 3 mL of liquid LB with 100 μg/mL of ampicillin. Then, plasmid DNA was extracted using the QIAprep Spin Miniprep Kit (Qiagen, Hilden, Germany, #27104) following the manufacturer’s protocol. After assessment of plasmid DNA integrity using Hind III (Promega, Madison, WI, USA, #R6041) and EcoRI (Promega, #R6011) restriction enzymes, antibiotic-resistant bacterial colonies were allowed to grow on a shaking platform overnight at 37 °C in 500 mL of liquid LB with 100 μg/mL of ampicillin. The day after, plasmid DNA was extracted using the QIAGEN Plasmid Maxi Kit (Qiagen, #12162) using the manufacturer’s protocol. Then, extracted plasmid DNA was quantified using Nanodrop ND-1000 technology.

Two different immortalization methods were tested: one based on liposomes and one mediated by electroporation. To reduce culture heterogeneity, CpBCs after seven passages in culture were employed for immortalization experiments.

For liposome-mediated transfection, one well of a 6-well standard plate of CpBCs at nearly 100% confluence was enzymatically detached using Accutase and seeded (1:2–1:4) in new 6-well standard plates. Upon reaching 80% confluence, CpBCs were transfected using Lipofectamine™ 3000 transfection reagent with either plasmid DNA encoding SV40 or hTERT or a combination of these latter two, following the manufacturer’s protocol. Lipofectamine™ 3000 was removed after 24 h. One 6-well plate each of CpBCs transfected with either a GFP-expressing plasmid (Monster Green^®^ Fluorescent Protein Vector, Promega, #E6421) or incubated with transfection reagent only was included to evaluate transfection efficiency and cytotoxicity through estimation of GFP-positive CpBCs using a fluorescence microscope and evaluation of cell recovery (e.g., attachment at the bottom of the plate) the day following electroporation, respectively.

For electroporation-mediated transfection, one well of a 6-well standard plate of CpBCs and CiBCs at nearly 100% confluence was enzymatically detached using Accutase and seeded (1:2–1:4) in new 6-well standard plates. Upon reaching 100% confluence, cells were enzymatically detached using Accutase and centrifuged at 400× *g* for 5 min. Cells were washed two times with PBS and resuspended in a 4 mm cuvette in 80–145 µL of Opti-MEM™ (Thermo Fisher Scientific, #31985062) at a cell density of 5–10 × 10^6^ cells/mL. Cells were then transfected with 3–8 μg each of plasmid DNA encoding either SV40 or hTERT, alongside a GFP-expressing plasmid using the ECM^®^630 Electroporation System (BTX, Harvard Apparatus, Holliston, MA, USA) and applying the following electroporation parameters: 280–500 V, 50 uF, 129–1575 Ω, and 1–3 pulses. After electroporation, cells were incubated for 5 min on ice, and media were changed the day after. Negative controls were included to assess cell viability.

One week post-transfection, CpBCs were subjected to selection with 350 µg/mL of G418 antibiotic (Merck, #G8168-10ML) in cBCsm3 for four weeks to establish CiBCs.

### 2.4. Protein Extraction and Western Blot of Chiropteran Primary and Immortalized Brain Cells

To characterize CpBCs and CiBCs, antibodies targeting phenotypic markers were selected based on the orthology of the targeted antigens to chiropteran proteins. The orthologous sequences were identified as the reciprocal best hits in GenBank through BLASTp v2.17.0 searches [63]. Sequences with E-value = 0.0 and 100% coverage were aligned with CLUSTALW to assess the conservation level [64]. Proteins showing ≥65% sequence identity were therefore selected for Western blot (WB) (Table 1). To reduce culture heterogeneity, CpBCs after seven passages in culture were employed for WB experiments.

For protein extraction, one well each of a 6-well standard plate of CpBCs and CiBCs at nearly 100% confluence was enzymatically detached using Accutase and seeded (1:2–1:4) in new 6-well standard plates. Upon reaching nearly 100% confluence, cells were lysed for 5 min using 120 µL of Pierce™ Ripa buffer (Thermo Fisher Scientific, #89901) supplemented with cOmplete Mini EDTA-free protease inhibitor cocktail tablets (Roche, #11836170001) following the manufacturer’s protocol. Cell lysates were then centrifuged at 4 °C at 14,000× *g* for 15 min. Protein concentrations were calculated using a Pierce™ BCA protein Assay kit (Thermo Fisher Scientific, #23225) following the manufacturer’s protocol.

For WB, 15 μg of proteins were first denatured at 70 °C for 10 min or at 95 °C for 5 min and then resolved using Bolt™ Bis-Tris Plus Mini protein gels, 4–12% (Thermo Fisher Scientific, # NW04125BOX), or NuPAGE™ Tris-Acetate Mini protein gels, 3–8% (Thermo Fisher Scientific, # EA03755BOX), and transferred to a nitrocellulose membrane using the iBlot™ Transfer Stack kit (Thermo Fisher Scientific, # IB301002). To verify equal protein loading and transfer efficiency, membranes were stained with AdvanStain Ponceau (Aurogene, Rome, Italy, #R-03021-D50) for 5 min at room temperature. To block non-specific binding sites, blots were incubated for 90 min in 5–10% non-fat dry milk in Tris-buffered saline (TBS) containing 0.05–2% Tween-20 (TBS-T) at room temperature. Then, blots were incubated at 4 °C overnight with selected primary antibodies diluted in TBS-T containing 1–5% non-fat dry milk (Table 1). To improve signal detection, several protocols were tested.Then, membranes were incubated with goat anti-rabbit IgG (H + L) poly-HRP secondary antibody (1:3000, Thermo Fisher Scientific, #32260) or goat anti-mouse IgG (H + L) poly-HRP secondary antibody (1:3000, Thermo Fisher Scientific, #32230) diluted in TBS-T for 1 h at room temperature. Reactive bands were visualized using the SuperSignal™ West Pico PLUS Chemiluminescent Substrate kit (Thermo Fisher Scientific, #34577). Protein extracted from canine tissues (brain, spleen, and smooth muscle), canine mammary tumor cells (Cellosaurus, Geneva, Switzerland, CIPp, #CVCL_L149), and dolphin immortalized fibroblast cells (patent n°102020000003248; https://www.unipd.it/en/brevetti/scheda/sea-sentinel-system-studio-ambiente) were included as positive controls.

### 2.5. Immunofluorescence of Chiropteran Primary and Immortalized Brain Cells

For immunofluorescence (IF), only the antibodies that showed binding to the expected-size proteins upon WB were investigated. To reduce culture heterogeneity, CpBCs after seven passages in culture were employed for IF experiments. One well of a 6-well standard plate of CpBCs and CiBCs at nearly 100% confluence was enzymatically detached using Accutase and seeded (1:2–1:4) in new 24-well standard plates. Upon reaching 60% confluence, cells were fixed with 4% paraformaldehyde solution for 20 min at room temperature. Cells were then washed with PBS containing 0.05% Tween-20 (PBS-T) and permeabilized with PBS containing 0.1% Triton X-100 for 15 min at room temperature. To block non-specific binding sites, cells were incubated with PBS containing 4% bovine serum albumin for 1 h at room temperature and then incubated at 4 °C overnight with selected primary antibodies (Table 1). Several protocols were tested.Cells were then incubated with goat anti-mouse IgG (H + L) Alexa Fluor^®^ 647 (1:200–1:1000, Aurogene, #AB150115-500UG) for 1 h at room temperature in the dark. Nuclei were counterstained with Hoechst33342 for 10 min (Thermo Fisher Scientific, #62249) and washed with PBS before imaging using the THUNDER Imager 3D Assay (Leica, Wetzlar, Germany).

### 2.6. RNA Extraction and Quantification of Chiropteran Primary and Immortalized Brain Cells

To reduce culture heterogeneity, CpBCs after seven passages in culture were employed for transcriptomic experiments. For RNA extraction, one well of a 6-well standard plate of CpBCs and CiBCs at nearly 100% confluence was enzymatically detached using Accutase and seeded (1:2–1:4) in new 12-well standard plates (three biological replicates). Upon reaching nearly 100% confluence, cells were enzymatically detached using Accutase and centrifuged at 400× *g* for 5 min. The obtained cell pellet was snap frozen at −80 °C until acid nucleic extraction was performed using the miRNAeasy Micro kit (Qiagen, #217084) following the manufacturer’s protocol. Extracted RNA was then quantified using both Nanodrop ND-1000 technology and the Qbit™ RNA BR Assay kit (Thermo Fisher Scientific, #Q10211). Samples were sent for RNA-sequencing (RNA-seq) to Biomarker Technologies (BMK) GmbH (Münster, Germany).

### 2.7. Bioinformatic Analyses

A total of six RNA-seq libraries derived from chiropteran brain cells were processed using the nf-core/rnaseq pipeline v3.22.0 (https://nf-co.re/rnaseq/3.23.0/). Sequencing reads were aligned to the *Eptesicus fuscus* reference genome (NCBI assembly GCA_027574615.1) using STAR. Gene-level quantification was performed with RSEM, generating raw read counts for each gene. The resulting gene-level count matrix was analyzed using iDEP v2.4.4. Lowly expressed genes were filtered out by retaining those with counts ≥0.5 counts per million in at least one sample. Data normalization and transformation were performed using the EdgeR pipeline with a pseudocount of 4 to stabilize variance. Missing values were imputed using the gene median approach. Gene identifiers were mapped to Ensembl/STRING databases, and differential expression analysis (DEG) was conducted using DESeq2 with the standard Wald test and independent filtering enabled. Genes were considered differentially expressed if they met a false discovery rate (FDR) threshold of 0.05 and a minimum fold-change (FC) of 2. Functional enrichment analysis was performed using Gene Set Enrichment Analysis (GSEA, pre-ranked mode) across Gene Ontology Biological Process (GOBP), Molecular Signatures Database Hallmark (MSigDB Hallmark), and Kyoto Encyclopedia of Genes and Genomes (KEGG) set collections, with pathway sizes restricted to 5–2000 genes and an FDR cutoff of 0.1. Directionality of FC was preserved. Similarly, to identify significantly enriched cellular signatures, a custom-made Signature database was realized using gene expression data obtained from the DropViz resource v2018.04.01 (http://dropviz.org/), based on the single-cell transcriptomic atlas of the adult mouse brain [65] (Table S1).

## 3. Results

### 3.1. DNA Barcoding Analysis Revealed Species Identification of Hypsugo savii

DNA barcoding analysis of the mitochondrial COI gene sequences revealed a high degree of similarity (>99%) with reference sequences of *Hypsugo savii* (Savi’s pipistrelle, Bonaparte, 1837), allowing the assignment of the sampled specimens to this species.

### 3.2. cBCsm3 Cell Culture Media Enables the Growth of Chiropteran Primary Brain Cells

**Figure 1:**
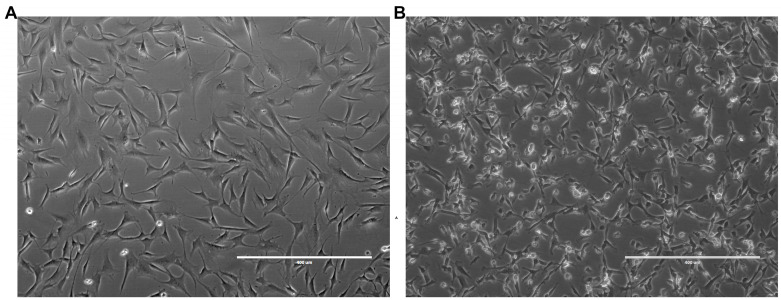
Brightfield images of chiropteran primary brain cells and chiropteran immortalized brain cells. (**A**) 50% confluent chiropteran primary brain cells, displaying a heterogeneous but mainly elongated fibroblast-like morphology; (**B**) nearly 50% confluent chiropteran immortalized brain cells, displaying a homogeneous but mainly elongated fibroblast-like and compact morphology (10×). Scale bar: 400 μm.

The day following plating, undigested tissue fragments were observed in both wells, sP1. After 72 h and five days following dissociation, CpBCs displaying a heterogeneous but mainly elongated fibroblast-like morphology began to adhere to sP1 and sP2 wells, respectively (Figure 1A). Eleven days after tissue dissociation, when CpBCs in both sP1 and sP2 cultures had reached approximately 100% confluence, only one well of sP2 was switched to cBCsm1. Within this well, after 24 h of cBCsm1 exposure, spheroidal aggregates resembling neurospheres emerged. However, these formations exhibited signs of cellular suffering, leading to a medium change back to cBCsm3 after six days. Four days after the introduction of cBCsm3, CpBCs in sP1 cultures again reached approximately 100% confluence and were further expanded, as previously described.

### 3.3. Simian Virus 40 Large T Antigen Mediates the Immortalization of Chiropteran Primary Brain Cells

After transfection with either pSV3-neo or pCl neo-hEST2, CpBCs showed different morphology, having a more ovaloid shape mixed with cellular debris when transfected with only the latter one, in comparison with the negative control. Similarly, when transfecting both plasmids at the same time, increased signs of cell suffering were observed; thus, the immortalization protocol was carried out only for pSV3-neo. At first, Lipofectamine™ 3000 was used following the manufacturer’s protocol, replacing the exhausted medium after 24 h. Due to no detection of GFP-positive cells in the controls, only the optimization of electroporation conditions was carried out.

Several protocol parameters were systematically optimized. Cell density and DNA concentration were adjusted to 7.5 × 10^6^ cells/mL and 8 µg of each plasmid DNA per reaction, respectively. CpBCs were resuspended in the minimum 80 µL volume recommended by the manufacturer for the 4 mm cuvette in order to maximize conductivity. Resistance (1575 Ω) and capacitance (50 µF) were set to achieve the longest possible pulse duration. Standard voltage conditions (280 V), reported in the literature, for mouse primary brain cell electroporation did not yield detectable GFP-positive CpBCs. Therefore, the voltage was increased to 500 V, resulting in optimal transfection efficiency. Having defined all the previous parameters, the number of electroporation pulses was set to two, as this condition provided the best balance between transfection efficiency and cell viability. Additionally, electroporated CpBCs were incubated on ice for 5 min to facilitate membrane pore stabilization and increase plasmid DNA uptake.

One week post-transfection, CpBCs were subjected to selection with 350 µg/mL of G418 cBCsm3 for four weeks to establish CiBCs. After six days, CiBCs-resistant colonies started appearing, showing a more homogeneous and elongated fibroblast-like morphology and growing in more compact cell colonies compared to CpBCs (Figure 1B). After four weeks, with a cell viability rate of approximately 50%, CiBCs were expanded and cryopreserved as previously described, with a splitting ratio between 1:10 and 1:20 according to planned experiments.

### 3.4. Chiropteran and Immortalized Brain Cells Express a Mesenchymal Marker by Western Blot

**Figure 2:**
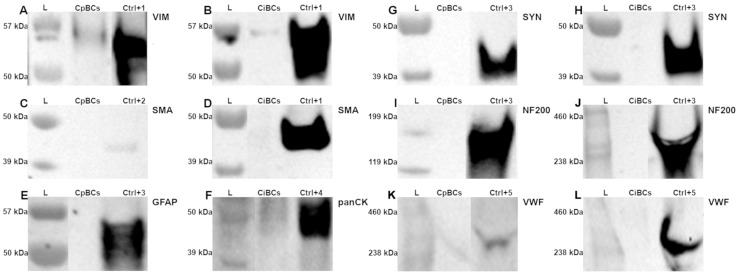
Western blot analysis of protein lysates of chiropteran primary brain cells and chiropteran immortalized brain cells. (**A**) Western blot expression of vimentin in chiropteran primary brain cells; (**B**) Western blot expression of vimentin in chiropteran immortalized brain cells; (**C**) Western blot expression of alpha-smooth muscle actin in chiropteran primary brain cells; (**D**) Western blot expression of alpha-smooth muscle actin in chiropteran immortalized brain cells; (**E**) Western blot expression of glial fibrillary acidic protein in chiropteran primary brain cells; (**F**) Western blot expression of pan-keratin in chiropteran immortalized brain cells; (**G**) Western blot expression of synaptophysin in chiropteran primary brain cells; (**H**) Western blot expression of synaptophysin in chiropteran immortalized brain cells; (**I**) Western blot expression of neurofilament 200 in chiropteran primary brain cells; (**J**) Western blot expression of neurofilament 200 in chiropteran immortalized brain cells; (**K**) Western blot expression of Von Willebrand factor in chiropteran primary brain cells; (**L**) Western blot expression of Von Willebrand factor in chiropteran immortalized brain cells, related to Figure S1. L: ladder; CpBCs: chiropteran primary brain cells; Ctrl + 1: dolphin immortalized fibroblast cells; CiBCs: chiropteran immortalized brain cells; Ctrl + 2: canine smooth muscle; Ctrl + 3: canine brain; Ctrl + 4: canine mammary tumor cells; Ctrl + 5: canine spleen.

Of the two mesenchymal cell markers, only vimentin (VIM), a 54–57 kDa protein, was detected in protein extracts from both CpBCs (Figure 2A) and CiBCs (Figure 2B), while alpha-smooth muscle actin (SMA), a 42 kDa protein (Figure 2C,D), was not detected. Glial fibrillary acidic protein (GFAP) and pan-keratin (panCK), 52 and 40–56.5 kDa proteins marking glial and epithelial cells, respectively, were both not detected in the CpBCs (Figure 2E) and CiBCs (Figure 2F). Similarly, synaptophysin (Figure 2G,H), neurofilament 200 (Figure 2I,J), and Von Willebrand factor (Figure 2K,L), 40, 200, and 500–10,000 kDa proteins, respectively, were detected neither in the CpBCs nor in the CiBCs. All protocols were validated by the presence of positive controls. Full gel images are available in Supplemental Information (Figure S1).

### 3.5. Chiropteran and Immortalized Brain Cells Express a Mesenchymal Marker at Immunofluorescence

**Figure 3:**
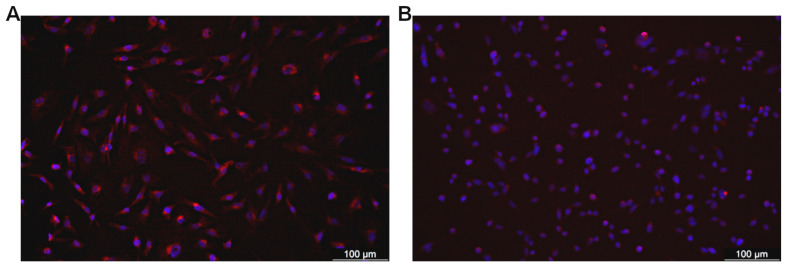
Immunofluorescence of chiropteran primary brain cells and chiropteran immortalized brain cells. (**A**) Immunofluorescence expression of vimentin in chiropteran primary brain cells; (**B**) immunofluorescence expression of vimentin in chiropteran immortalized brain cells (20×). Vimentin-positive staining is observed in red within the cytoplasm, while cell nuclei are counterstained in blue.

Mild and strong cytoplasmic positivity for VIM (Figure 3A,B) was detected in both CpBCs and CiBCs, respectively.

### 3.6. Chiropteran Immortalized Brain Cells Show Activation of Cell Cycle Mitotic Pathways and a Less Heterogeneous Cell Composition Compared to Chiropteran Primary Brain Cells

**Figure 4:**
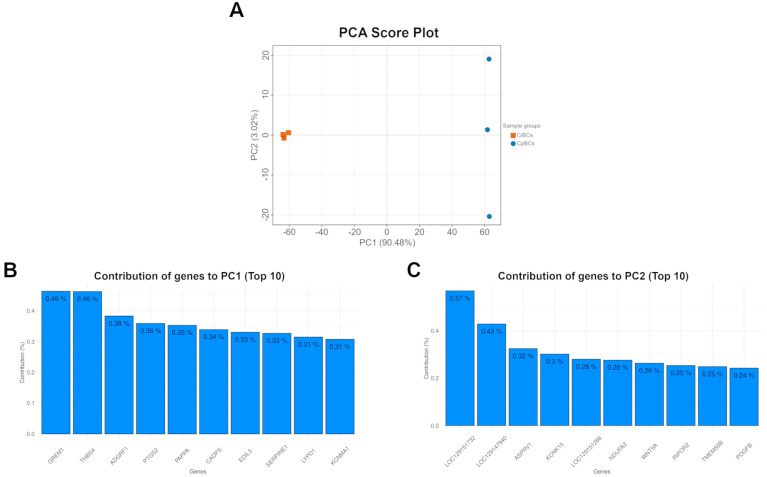
Differential expression analysis summary plots. (**A**) Principal component analysis showing the primary and secondary components of the total variance between chiropteran primary brain cells (blue circles) and chiropteran immortalized brain cells (red squares); (**B**) histogram showing the genes contributing the most to the primary component of the total variance; (**C**) histogram showing the genes contributing the most to the secondary component of the total variance. X-axis: gene names; y-axis: contribution (%).

Following preprocessing, 25,780 unique genes were detected across the samples, of which 12,933 passed the filtering criteria and were retained for downstream DEGs. An initial unsupervised analysis was conducted by means of principal component analysis (PCA) which demonstrated that the primary source of the total variance (PC1, 90.48%) segregated CiBCs from CpBCs, whereas the secondary remaining of the total variance (PC2, 3.02%) was attributable to intra-group differences (Figure 4A), driven by genes such as *ADGRF1*, *PTGS2*, *CADPS*, *EDIL3*, *LYPD1, KCNMA1*, *NDUFA3*, *TMEM50B*, and *PDGFB*, respectively (Figure 4B,C). DEGs revealed marked transcriptional differences between CpBCs and CiBCs with 3073 differentially expressed genes (1527 upregulated and 1546 downregulated). The full list of up- and downregulated genes is available in Supplemental Information (Table S2). Among the differentially expressed genes with the highest FC (i.e., upregulated), genes such as *ENPP3*, *MYRF*, *RASGRF1*, *CGN*, *ENO2*, *PLEKHG6*, *CELSR1*, *ENTREP1*, *P2RY2*, *ISG15*, *FGFR4*, *MAG*, *DUSP26*, *EFNB3*, *RASAL1*, and *LGI1*, were found, while genes such as *IL13RA2*, *ADGRF1*, *BRINP1*, *PCDH19*, *MMP1*, *NTM*, *NEFM*, *MMP13*, *KCNMA1*, *PCDH20*, *CDH6*, *GREM1*, *FRMPD4*, *CNTNAP2*, *OLFM4*, *THBS4*, *SEMA6A*, *RUBCNL*, *NPY*, *PAPPA*, *PTGS2*, *LYPD1*, *ZIC1*, *CDH13*, *CADPS*, *SRPX2*, *ADGRL4*, *EVI2A*, *SERPINE1*, *GRIK2*, *ALK*, *DIO2*, *NRP2*, *GRIA3*, and *VIM*, were found among the top downregulated genes in CiBCs compared to CpBCs.

**Figure 5:**
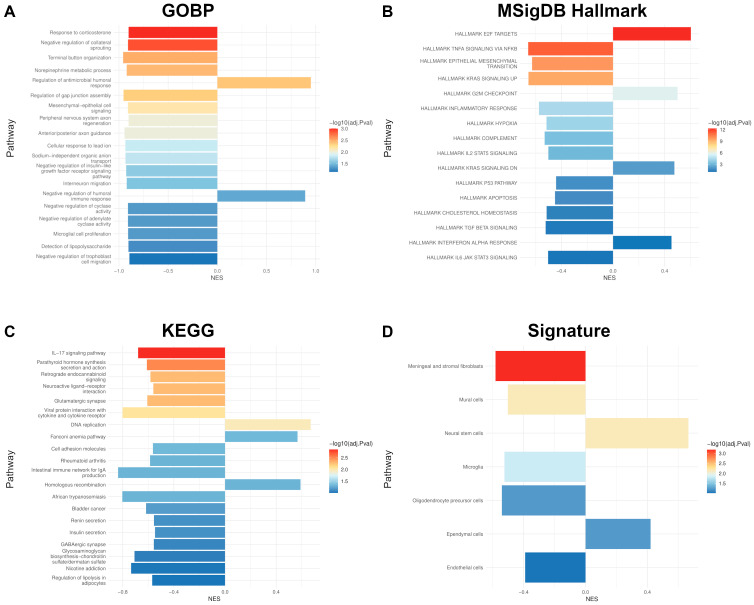
Gene Set Enrichment Analysis summary plots. (**A**) Histogram showing the Gene Set Enrichment Analysis resulting pathways run on the Gene Ontology Biological Process; (**B**) histogram showing the Gene Set Enrichment Analysis resulting pathways run on the Molecular Signatures Database Hallmark; (**C**) histogram showing the Gene Set Enrichment Analysis resulting pathways run on Kyoto Encyclopedia of Genes and Genomes; (**D**) histogram showing the Gene Set Enrichment Analysis resulting pathways run on the custom-made Signature database. X-axis: Normalized Enrichment Score (NES); y-axis: name of the pathway.

To gain insight into the different pathways between the two populations, GSEA, run on the GOBP database, was performed. GSEA revealed strong upregulation of the pathways associated with the regulation of antimicrobial humoral responses and negative regulation of humoral immune responses, alongside the downregulation of processes related to mesenchymal–epithelial cell signaling, negative regulation of the insulin-like growth factor receptor signaling pathway, adenylate cyclase activity, and microglial cell proliferation in CiBCs (Figure 5A). In a similar fashion, GSEA run on the MSigDB Hallmark database showed significant enrichment of proliferative and metabolic programs in CiBCs, including E2F targets, G2/M checkpoints, KRAS-downregulated pathways, and interferon alpha response, while TNFA signaling, epithelial–mesenchymal transition, KRAS-upregulated pathways, inflammatory response, p53, apoptosis and TGF-beta signaling were downregulated (Figure 5B). Similar results were also obtained when running GSEA against the KEGG database, which highlighted the upregulation of core pathways such as DNA replication and homologous recombination, alongside the downregulation of metabolic and signaling pathways, including IL-17-driven processes, viral protein interactions with cytokines and cytokine receptors, and cell adhesion molecules (Figure 5C). Complementary, GSEA against the custom-made Signature database revealed that brain cell-type-associated signatures were predominantly enriched in CpBCs compared to CiBCs, in which an NSC-like phenotype seemed to be predominant (Figure 5D). The full list of pathways resulting from the GSEA against GOBP, MSigDB Hallmark, KEGG, and the custom-made Signature database is available in Supplemental Information (Table S3).

## 4. Discussion

Recent pandemics and the continuing impacts of climate change have further highlighted the significance of the *One Health* approach, which recognizes the strong interdependence between human, animal, and environmental health [1,2,3]. Within this framework, bats (order *Chiroptera*) have emerged as key reservoir hosts for a wide range of zoonotic pathogens, while also attracting attention for their unique immunological traits, including modulated inflammatory responses and tolerance to viral infections [1,4,5]. These characteristics make them valuable models for studying both infectious and non-infectious diseases, particularly those affecting the brain [2]. Therefore, in our study, we challenged the isolation and establishment of CpBCs and CiBCs, with a specific focus on the analysis of transcriptomic changes underlying the immortalization process to gain a better insight into the reliability of these 2D-*in vitro* brain models.

Given the high species diversity within bats, accurate identification is essential not only for assessing species-specific zoonotic risk but also for understanding their distinct ecological roles [1,2,3]. In this study, DNA barcoding analysis identified the sampled individual as *Hypsugo savii* (Savi’s pipistrelle, Bonaparte, 1837). This species’ frequent roosting in anthropogenic structures facilitates closer proximity to humans and domestic animals [2,3,7]. Therefore, environmental pressures, including urbanization, habitat fragmentation, and climate change, may further influence its distribution, immune dynamics, and pathogen shedding patterns [1,2,3]. With particular regard to infectious diseases associated with this species, viruses such as Coronavirus, Issyk-Kul virus, and Lyssavirus are considered zoonotic, while other species-related pathogens, *Hexametra angusticaecoides*, *Hypsugopoxvirus*, and *Ixodes simplex*, have so far not been linked to human infection [7,8,9,10,66,67,68,69,70,71,72,73,74]. Additionally, *Hypsugo savii* parasites such as Diptera and Siphonaptera may act as mechanical or biological vectors of zoonotic agents under specific ecological conditions [68]. Hence, integrated surveillance approaches combining ecological, molecular, and epidemiological data with 2D-*in vitro* models of *Hypsugo savii* are essential to better understand host–pathogen interactions and spillover risks [2].

Regarding bat 2D-*in vitro* models, only one lung epithelial cell line (e.g., Tb 1 Lu) from the *Tadarida brasiliensis* species is commercially available, but several in-house isolation protocols have optimized the establishment of chiropteran primary and immortalized cells from several tissues, either from embryos or post-natal individuals [5,6,12,14,71]. To our knowledge, however, chiropteran brain cell lines still remain scarce, being the current ones reported only for some bat species, excluding *Hypsugo* spp. [75,76,77,78,79,80,81,82,83,84,85,86,87,88,89,90,91,92,93,94,95,96,97,98].

In this study, cerebral tissue was rapidly processed for cell isolation. Rapid brain tissue processing is important because of its abundance in lipids and intrinsic cell susceptibility to post-mortem lysis [99,100]. Several brain dissociation protocols have been reported both in human and other animal species, involving commercial kits, such as mechanical or enzymatic digestion employing the use of Accutase, Collagenase, Dispase, or Trypsin [92,99,100,101]. In the current literature in veterinary medicine, Papain digestion is the most effective strategy used for isolating viable cells from the brain, despite other studies’ enriched CpBCs using mechanical dissociation and Trypsin digestion [75,76,77,78,79,92,102]. Additionally, tissue filtration, delayed media change, and repeated transfer of the supernatants to fresh plates allowed the maximization of CpBCs recovery [53,54,99,101,103,104,105,106]. To enrich NSCs present in the adult mammal brain, because of their self-replicative tendency and extended lifespan, we tried culturing CpBCs with cBCsm1, given their reported tendency to form neurospheres when grown in media without serum [38,107]. However, increased cell suffering was noticed, leading to a return to cBCsm3 medium [38,51,99,100,101,107,108,109,110]. CpBCs showed heterogeneous but mainly elongated fibroblast-like morphology, as previously reported both in human and veterinary brain studies. Moreover, these findings seem to be in accordance with other isolated bat brain cell lines [75,76,77,89,92]. Accutase was used for cell detachment, and cBCsm3 was subsequently adopted as the maintenance medium based on established protocols for human and veterinary brain cells, despite the composition of the media for the culture of other CpBCs being less rich, and, in most cases, requiring only basal media and FBS [75,76,77,78,79,99].

In the literature, for continuous cell establishment, CpBCs were transfected with constructs encoding either SV40 or hTERT, targeting p53 suppressor pathways and the maintenance of telomere length, respectively [15,16,17,20,22,23,24,27,31,48,110,111,112]. In our study, CpBCs hTERT-mediated immortalization (in combination or not with SV40) resulted in increased cell suffering, whereas SV40-only transfected cells did not, as confirmed also by the current literature [55,76,77,89,102]. Interestingly, although the co-expression of SV40 and hTERT is often reported to enhance immortalization efficiency, hTERT-induced toxicity has been described in sensitive human cell types such as epithelial cells [20,24]. Different from our manuscript, other studies involving the immortalization of human, veterinary, and bat brain cells also reported the establishment of hTERT- and spontaneously immortalized CpBCs, highlighting a great variability across several *in vitro* systems [58,59,75,76,92]. Moreover, immortalization via liposome-mediated transfection was first approached in our study due to its relative simplicity, cost-effectiveness, and minimal technical requirements, although its efficiency strongly depends on the endocytic capacity of the target cells [15,16,32,33,34]. On the other hand, electroporation requires special equipment and more optimization, despite it being much more effective and useful to engineer hard-to-transfect cells, which could be a translatable characteristic to CpBCs, given their high resistance to pathogen entry [21,22,32,33,34]. Different from the immortalization of CiBCs carried out by He et al., in our study, no liposome-mediated transfection was achieved [77]. This might be due to either low endocytic activity, confirmed by resilience to a viral infection of chiropters, or a peculiar composition in the membrane lipids [15,16,32,33,34]. However, no further studies have described bat cell membrane characteristics so far. Despite Crameri et al. and Gonzalez et al. achieving the immortalization of CpBCs with retroviral and lentiviral transduction, in our study, we employed electroporation for the first time to avoid permanent genome integration and viral host-species specificity [76,102]. No morphological changes were previously reported in immortalized bat brain cells compared to primary cultures, despite the fact that, in our case and in other veterinary species, a less heterogeneous phenotype was observed [30,35,38,42,44,58,59,105].

Despite the high sequence homology between bat proteins and the epitopes targeted by the selected antibodies, both WB and IF analyses of CpBCs and CiBCs revealed expression exclusively of VIM, suggesting a mesenchymal phenotype [113,114]. However, the RNA-seq showed downregulated *VIM* expression in CiBCs compared to CpBCs, supporting the loss of cell-specific phenotypes due to oncogene-driven immortalization, also reported only in human studies [115,116]. Indeed, PCA revealed that immortalization was the dominant source of transcriptional variance, exceeding intra-group heterogeneity. Therefore, the immortalization process appeared to generate a more homogeneous and clonal population, likely due to selective pressure favoring a specific cell type capable of successfully transcribing the plasmid and surviving the oncogenic stress induced by antibiotic-based treatment despite the original heterogeneity of CpBCs. Supporting this hypothesis, CiBCs exhibited marked upregulation of genes, including *ENPP3*, *CGN*, *P2RY2*, *ISG15*, *FGFR4*, *RASAL1*, and *LGI1*, which are involved in pathways regulating cell proliferation and epithelial morphogenesis and have been reported to be enriched in brain cancers (e.g., glioma) [117]. Moreover, *DUSP26*, a gene implicated in glioblastoma progression through its ability to dephosphorylate and inactivate p53, was also upregulated, consistent with pathways expected to be specifically enriched in SV40-driven immortalization [117]. These findings, alongside the downregulation of genes that drive differentiation, apoptosis, and inflammatory and oncosuppressor mechanisms (e.g., *IL13RA2*, *BRINP1*, *GREM1*, *FRMPD4*, *OLFM4*, *SEMA6A*, *RUBCN*, *CDH13*, and *DIRAS3*), as well as the enrichment of the NSC-like signature in GSEA against our custom-made database, seem to support the partial dedifferentiation process in CiBCs compared to CpBCs, also reported in human studies [115,116,117,118,119,120,121,122]. Interestingly, GSEA that is run on the GOBP database showed negative regulation of the humoral immune response, suggesting a shift in suppressing inflammatory processes to avoid apoptosis and promote survival, while downregulated mesenchymal–epithelial signaling, negative feedback IGF, and microglial differentiation processes may be due to a loss in heterogeneity of CiBCs compared to CpBCs and uncontrolled proliferation mechanisms common in cancer cells [4,117,118,119,120,121,122]. Indeed, GSEA run on the MSigDB Hallmark and KEGG database confirmed the upregulation of cell cycle progression pathways, highlighting all the processes involving immune response, apoptosis, and p53 signaling as downregulated [4,118,119,120,121,122].

### Limitations of the Study

Tissue freshness and transport conditions likely affected pSCs yield and viability, highlighting the need for further improvement of sample handling strategies, such as immediate processing limiting time for transport. In addition, the neurogenic niche within the chiropteran brain remains poorly characterized, and more refined area-specific isolation approaches may be required to enrich NSCs. Further, although CiBCs clones survived antibiotic selection, G418 sensitivity assays could not be performed due to the limited replicative potential of CpBCs. Additionally, the genome of *Hypsugo savii* species was not yet available, so reads were therefore aligned to the genome of the most closely related species. Finally, to further validate the RNA-seq findings and determine whether the observed transcriptional changes associated with cell immortalization are representative at the species level, future studies should include DEGs of samples derived from multiple individuals.

Moreover, chiropteran positive controls and housekeeping target antibodies were not available, making the WB analysis specifically qualitative and not quantitative. Therefore, future studies should include comprehensive molecular characterization of CpBCs and CiBCs using approaches such as single-cell RNA sequencing and karyotype analysis to better define cellular identity and genomic stability.

Finally, as CpBCs and CiBCs represent 2D-*in vitro* culture systems, they cannot fully recapitulate the structural, cellular, or functional complexity of brain tissue *in vivo*. While these models provide valuable tools for mechanistic investigations, their limitations should be acknowledged. Future studies should therefore complement these systems with more physiologically relevant 3D-*in vitro* models and *in vivo* approaches to obtain a more comprehensive understanding of the biological processes involving these cells and to better assess their relevance within the native tissue context.

## 5. Conclusions

In conclusion, we successfully established the first brain-derived 2D-*in vitro* models from *Hypsugo savii*, including CpBCs and CiBCs, and characterized the molecular and phenotypic changes associated with their derivation and immortalization. Our findings indicate that SV40-driven immortalization promotes the selection of a more homogeneous, proliferative cell population, accompanied by transcriptional reprogramming toward cell cycle progression, partial dedifferentiation, and suppression of apoptosis and immune-related pathways. These features, together with the enrichment of cancer-associated and NSC-like signatures, highlight both the utility and the limitations of CiBCs as experimental models, particularly when investigating physiological versus transformation-associated processes. Importantly, the establishment of brain-derived cells from *Hypsugo savii*, a widespread synanthropic bat species in Europe, can provide a novel and regionally relevant platform for studying processes such as neurotropism, host–pathogen interactions, and species-specific immune dynamics, reinforcing the importance of integrating bat-derived 2D-*in vitro* systems within a *One Health* framework [1,2,3].

## Figures and Tables

**Table 1:** Primary antibodies used in Western blot and immunofluorescence to characterize chiropteran primary brain cells and chiropteran immortalized brain cells.

Antibody Company, Code (Clone)	Host Species	Targeted Antigen	Molecular Weight	Western Blot Dilution	Immunofluorescence Dilution
Dako (Glostrup, Denmark), M7315 (DAK-SYNAP)	Mouse	Human synaptophysin	40 kDa	1:500	N/A ^1^
Dako, M3515 (AE1/AE3)	Mouse	Human pan-keratin	40–56.5 kDa	1:800	1:25
Dako, M0851 (1A4)	Mouse	Human alpha-smooth muscle actin	<42 kDa	1:1000	1:100
Diagnostic BioSystems (Pleasanton, CA, USA), MOB199-05 (6F2)	Mouse	Human glial fibrillary acidic protein	52 kDa	1:1000	1:50
Dako, M0725 (V9)	Mouse	Porcine vimentin	54–57 kDa	1:5000	1:50
Merck, N0142 (N52)	Mouse	Human neurofilament 200	200 kDa	1:1000	N/A ^1^
Dako, A0082 (polyclonal)	Rabbit	Human Von Willebrand factor	500–10,000 kDa	1:1000	N/A ^1^

^1^ N/A: not applicable.

## Data Availability

Cell lines generated in this study will be made available on request, but we may require a payment and/or a completed materials transfer agreement if there is potential for commercial application. RNA-sequencing data have been deposited in the NCBI SRA database as PRJNA1466837 and are publicly available as of the date of publication. This paper does not report original code. Any additional information required to reanalyze the data reported in this paper is available from the lead contact upon request.

## Supplementary Materials

The following supporting information can be downloaded at: https://www.mdpi.com/article/10.3390/ani16132037/s1, Figure S1: Full Western blot gel pictures for the characterization of chiropteran primary brain cells and chiropteran immortalized brain cells, related to Figure 2. * = other primary antibody stained on the same membrane (to be ignored); # = aspecific signal; Table S1: Excel table of gene expression data obtained from the DropViz resource, based on the single-cell transcriptomic atlas of the adult mouse brain; Table S2: Excel table of the up- and downregulated genes in CiBCs compared to CpBCs, their FC and FDR; Table S3: Excel table of the GSEA enriched pathways across GOBP, MSigDB Hallmark, KEGG and custom-made Signature database.

## Abbreviations

The following abbreviations are used in this manuscript:

NS	Nervous system
pSCs	Primary somatic cells
SV40	Simian virus 40 large T antigen
TERT	Human telomerase reverse transcriptase
NSCs	Neural stem cells
CpBCs	Chiropteran primary brain cells
CiBCs	Chiropteran immortalized brain cells
COI	Cytochrome c oxidase subunit I
PBS	Phosphate-buffered saline
P/S	Penicillin–streptomycin
sP	Standard plate
DMEM/F-12	Dulbecco’s Modified Eagle Medium/Nutrient Mixture F-12
Glutamax	GlutaMAX™ supplement
NEEA	MEM Non-Essential Amino Acids solution
N-2	N-2 supplement
B-27	B-27™ supplement
hFGF	Human basic fibroblast growth factor
hEGF	Human epidermal growth factor
FBS	Fetal bovine serum
WB	Western blot
TBS	Tris-buffered saline
TBS-T	Tris-buffered saline containing 0.05–2% Tween-20
IF	Immunofluorescence
FDR	False discovery rate
FC	Fold-change
GOBP	Gene Ontology Biological Process
MSigDB Hallmark	Molecular Signatures Database Hallmark
KEGG	Kyoto Encyclopedia of Genes and Genomes
VIM	Vimentin
SMA	Alpha-smooth muscle actin
GFAP	Glial fibrillary acidic protein
panCK	Pan-keratin
SYN	Synaptophysin
NF200	Neurofilament 200
VWF	Von Willebrand factor
PCA	Principal component analysis
NES	Normalized Enrichment Score
