# Supplementary figures and images for: Chiropteran (Hypsugo savii) Post-Natal Brain 2D-In Vitro Models: Primary Cell Isolation, Immortalization and Transcriptomic Changes
Source: Animals (Basel). 2026 Jul 2;16(13):2037. doi: 10.3390/ani16132037 (PMC13360592; doi:10.3390/ani16132037)

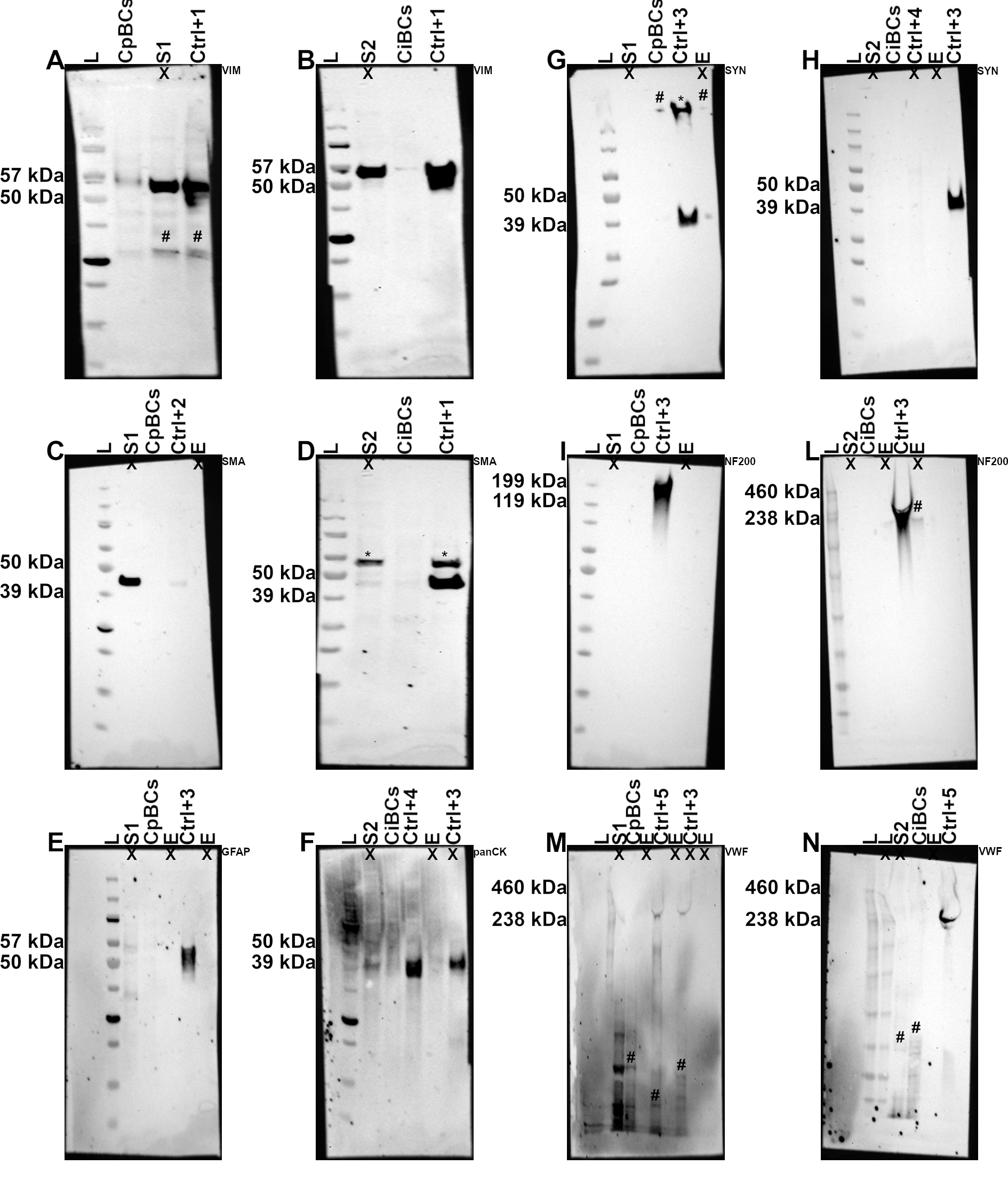

Supplement: Supplementary file 1 [file animals-16-02037-s001.zip › Figure S1.tif]
